# Role of the Fas/FasL pathway in HIV or SIV disease

**DOI:** 10.1186/1742-4690-6-91

**Published:** 2009-10-15

**Authors:** Bhawna Poonia, C David Pauza, Maria S Salvato

**Affiliations:** 1 Institute of Human Virology, University of Maryland, School of Medicine, 725 W Lombard Street, Baltimore, MD 21201, USA

## Abstract

Human immunodeficiency virus disease involves progressive destruction of host immunity leading to opportunistic infections and increased rates for malignancies. Both depletion in immune cell numbers as well as defects in their effector functions are responsible for this immunodeficiency The broad impact of HIV reflects a similarly broad pattern of cell depletion including subsets that do not express viral receptors or support viral replication. Indirect cell killing, the destruction of uninfected cells, is due partly to activation of the Fas/FasL system for cell death. This death-signaling pathway is induced during HIV disease and contributes significantly to viral pathogenesis and disease.

## Background

Changes in CD4 cell count and viral RNA burden are the most commonly used markers for HIV disease progression. However, evidence has existed for several years that many patients with HIV disease experience a broad loss of leukocyte subsets without an apparent preference for depleting CD4 T cells [1-3]. Effects on cell types other than CD4 T cells were documented in macaques after showing substantial B cell loss during acute SIV infection [4] and in humans by showing depletion of γδ T cells [5] that are CD4 negative. Many other examples confirmed that the profound impact of HIV on "non-CD4" leukocyte populations must depend on indirect mechanisms, as opposed to direct cell killing that occurs when HIV or SIV infects and destroys susceptible CD4+ cells. Uninfected CD4+ cells can also be destroyed by indirect mechanisms. Since both direct and indirect mechanisms are driven by viral burden, it has been difficult to distinguish their contributions to CD4+ T cell depletion and progressing disease. This technical obstacle has blocked efforts to explore new therapies that target indirect mechanisms.

**Figure 1:**
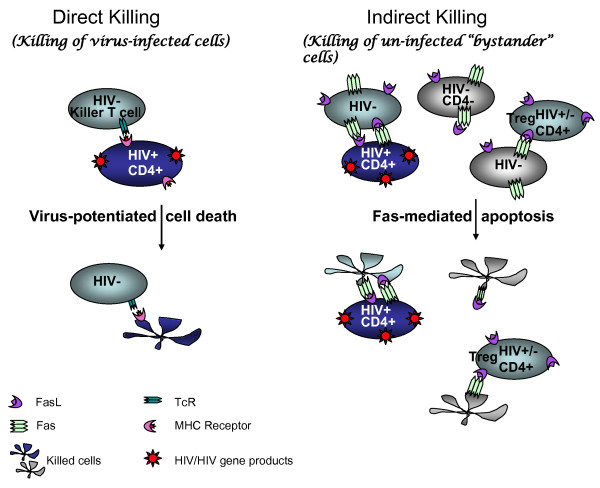
**Major mechanisms of leukocyte cell loss in AIDS**. Two models for cell death in AIDS are the direct and indirect killing of leukocytes during disease progression. Direct killing, or killing of virus-infected cells, is presumed to be virus-mediated or to occur via immune surveillance of virus-infected cells, most often by killer T cells. The virus-infected cells are predominately memory T cells with the phenotype CD4+CD45RA- or CD4+CD45RA+Fas and are primarily killed by cytotoxic T cells in a Fas-independent manner [52]. Indirect cell death, or killing of uninfected "bystander" cells, has also been documented *in vivo*. All leukocytes, including uninfected bystander cells, can be activated, with up-regulation of Fas/FasL and other death mediators, after contact with HIV-infected cells or HIV antigens such as soluble *tat, gp120, vpr*, and *nef *[20,24,38,39,49]. Thus HIV gene expression contributes to both direct and indirect killing mechanisms. Contact with death ligands like FasL causes apoptosis of activated cells through Fas/FasL signaling. Tregs are major effectors of bystander killing. The finding that HIV+ cells are less susceptible to Fas/FasL killing means that HIV+ cells become enriched when Fas-mediated apoptosis is the major death pathway.

Besides depletion of immune cells due to cell death, HIV infection is also characterized by defects in effector cell functions. T cell exhaustion or low HIV-specific T cell cytotoxicity has been attributed to loss of zeta chains [6] or to high levels of PD-1 or CTLA-4 on the surface of T cells [7,8]. During chronic HIV infection this immune dysfunction can result from interactions with regulatory T cells (Tregs) [9,10] that are exported to the periphery during high thymic turnover [11]. Ultimately, Tregs contribute not only to immune exhaustion but also to cell death via the Fas/FasL apoptotic pathways (Fig. 1).

### Mechanisms of indirect cell depletion in HIV infection

Despite overwhelming evidence for indirect cell depletion, little is known about how it occurs *in vivo*. Cell loss is accelerated during elevated viremia and a broad recovery of leukocytes attends highly active antiretroviral therapy (HAART) [12]. Some of these changes may be related to release of sequestered lymphocytes from secondary lymphoid tissues [13] as viral antigen declines during therapy. HIV effects on bone marrow, thymus, and hematopoietic stem cells were also proposed as causes for general leukocyte loss [14]. While HIV does indeed infect and alter bone marrow and thymus, the kinetics for cell loss during acute infection, the rates for recovery during HAART and the durable repertoire defects in T and B cell subsets after HAART [15,16] argue that these dynamic changes occur within mature populations without an overwhelming impact of declining stem cell output. Therefore due to large expansions and contractions of mature cell numbers, declining stem cells do not have a measurable impact on leukocyte depletion in AIDS.

A process described as "chronic immune activation" or "hyperactivation" occurs during HIV disease and is accompanied by higher expression of TNF superfamily ligands and their receptors, e. g. Fas/FasL and TRAIL-DR5 [17-20]. Chronic activation coincides with "activation induced cell death" (AICD) that was initially coined to describe cell loss that occurs when lymphocytes are activated by viral antigen in the absence of appropriate costimulation. AICD connects viral gene expression to a general lymphocyte destruction, ultimately resulting in reduced immune protection from HIV. It is now known that both viral and host products can activate lymphocytes and induce death receptor expression. Secreted HIV Tat protein has been shown to up-regulate FasL and TRAIL in T cells or macrpophages, which can in turn induce apoptosis in bystander cells, thereby providing a mechanism of cell death in uninfected cells [21-23]. In HIV disease, both antigen-specific and polyclonal activation have been observed [17,24,25]. This pan-activation sensitizes the lymphocytes to apoptotic cell death that occurs when the death ligand, usually cell-associated, contacts a cell that is expressing death receptors.

Early investigators [26] proposed apoptotic death as an important contributor to CD4 T cell depletion. They observed that triggering through the T cell receptor failed to induce proliferation of PBMC from HIV+ asymptomatic donors; instead, CD4 cells in these cultures had features consistent with Fas-mediated apoptosis [27]. We confirmed this result in SIV-infected macaques [28]. Alternatively, signaling through the T cell receptor [29] can activate the mitochondrial pathway for apoptosis, distinct from the Fas-triggered caspase-8 death pathway [30]. Both pathways are generally invoked in AICD (Figure 1). Since antigen-specific memory T cells are more likely to express Fas, cell killing can appear to be tied to antigen specificity. Both the caspase 8 and the mitochondrial apoptosis mechanisms tend to delete immune cells that respond to antigen. Preferential apoptotic cell death of antigen-activated T cells could account for the lack of immune control over opportunistic pathogens and an insufficiency of viral immunity that fails to prevent viral persistence or progressing disease. Once the host environment is "set," and after viremia triggers higher expression of FasL, every encounter with antigen has the potential to drive cell depletion among all lymphocyte compartments.

### Role of Fas/FasL mediated cell death in HIV/SIV infections

Apoptosis is observed consistently among uninfected cells in SIV+ macaques or HIV+ humans [31]. Chronic immune activation drives cells into apoptosis [32] possibly involving Fas/FasL interactions [33], and reflects an exaggeration of the normal processes for homeostatic cell regulation during HIV disease [34]. The roles of assassin and victim are not always clear in these interactions. Activated CD4+ T cells can express FasL and become the effectors of cell death including the destruction of resting B cells [35], even as they also become targets for cell killing and display high rates for apoptosis during disease.

Many studies have explored the mechanisms connecting HIV/SIV antigen expression and apoptotic cell death. Human macrophages express FasL after exposure to HIV [36], creating a link between antigen presentation and Fas/FasL-mediated apoptosis. HIV up-regulates FasL in CD4 T cells [37,38] after they are exposed to soluble Tat, gp120 [22] or Nef proteins [39]. Higher levels of FasL, both cell-associated and in plasma, and Fas were observed in specimens from HIV+ patients whose PBMC were especially susceptible to Fas-mediated cell death *in vitro *[40]. Cross-linking of CD4 by gp120 complexes or viral particles increased the susceptibility to apoptosis triggered by FasL or TNF-α [41,42].

The patterns of CD4 T cell depletion appear to be antigen-specific, as perturbation of the CD4 receptor repertoire is significantly associated with higher plasma viremia [43]. This can be explained by an AICD mechanism or by virus infection of antigen-activated cells as we proposed [44]. Less data are available to show whether CD8+ T cell or B cell loss during HIV infection is antigen-specific, although altered receptor repertoire have been reported in both cases [45-47]. The γδ T cell population shows a specific pattern of depletion in HIV disease [5], losing the critical Vγ2-Jγ1.2+ subset that is required for pathogen and tumor cell responses but few cells express CD4 and γδ T cells do not support HIV replication. To the extent that antigen stimulation is related to cell depletion, AICD may be invoked as a mechanism for γδ T cell killing. Thus, AICD is a mechanism that potentially links antigen stimulation with expression of death ligand receptors, leading to specific cell depletion.

Neuronal cells that are depleted during chronic HIV infection comprise an important example of bystander depletion since neuronal cells are not infected by HIV. Neuronal cell loss has been attributed to interaction with viral proteins such as gp120, vpr, nef, and tat, and to soluble neurotoxic factors released by infected macrophages [48]. The primary mechanism of neuronal depletion is apoptosis via extrinsic Fas-related death receptors or intrinsic mitochondrial pathways [48]. The fate of neuronal cells during AIDS is reminiscent of the cell culture studies previously mentioned documenting the apoptotic destruction of uninfected cells exposed to viral proteins [20,24,38,39,49].

Broadly acting immune stimulation during HIV or SIV infection especially implicates the Fas death pathway since activated T or B cells express Fas. Acute SIV infection triggers rapid increases in Fas and FasL expression in peripheral blood [50] as well as in thymus and other lymphoid tissues [51]. During acute SIV infection, most T cells express Fas [52] and there is abundant local expression of FasL among intestinal lymphocytes [53]. Ablation of intestinal lamina propria CD4+ cells during SIV infection can be attributed in part, to the Fas/FasL mediated apoptotic pathway but it is controversial whether apoptotic death of uninfected cells exceeds virus-mediated killing of infected cells. Detailed studies of viral burden and the rising proportion of SIV+ intestinal T cells argued that direct depletion of infected cells accounts for the rapid cell loss [52] without the need to invoke apoptotic killing of uninfected cells. These investigators stated that up to 60% of all memory T cells were infected but that those cells were lost within 4 days. They defined memory CD4+ cells as those that were CD45RA- or CD45RA+CD95+, meaning that Fas+ (CD95+) cells that are highly susceptible to apoptosis are included. Circulating lymphocytes and lymphocytes from lymph nodes and mucosa were sampled, but lymphocytes were not sampled from the spleen or liver, for example, so it is impossible to rule out margination as an explanation for lymphocyte loss. There is no way to determine from the experimental design, whether infected cells are being depleted faster than uninfected cells.

Another recent publication from Mattapallil's laboratory notes that early mucosal HIV/SIV infection (2-4 days after inoculation) there are high level of infected CD4+ TH-17 T cells that are depleted during the course of infection [54]. TH-17 cells are proinflammatory effectors of antiviral immunity and are commonly suppressed by Treg, most likely causing their depletion via the Fas pathway. Once again it is not clear whether this depletion is due to migration or cell death in the category of direct depletion of virus-infected cells or indirect depletion of uninfected bystander cells. It is highly improbable that any one mechanism will explain all events in AIDS pathogenesis.

Several important studies have implicated apoptosis in uninfected cells as a major mechanism for leukocyte depletion. Fas ligation was a probable cause of apoptosis in T cells from SIV infected macaques [55]. However, caspase-independent pathways for T cell apoptosis were thought to drive cell death in other SIV infection studies [56]. Cell loss, especially in gut-associated lymphoid tissues, likely occurs by multiple mechanisms and we would expect depletion of both CD4+ and CD4- cell subsets at these loci of intense viral replication.

A recent publication describes gene expression profiles of three stages of HIV infection: acute, chronic, and AIDS [57]. The acute stage has high levels of FasL mRNA expression that diminishes during the chronic stage. This observation coincides with earlier published studies showing a high level of PBMC-susceptibility to AICD during the acute stage of SIV infection, and this susceptibility subsides during the chronic stage [58].

Curiously, infected cells are more resistant to apoptosis than uninfected cells [23,59]. The apoptosis resistance in persistently infected lymphoid and monocytic cells was shown to be independent of active viral production and involved a modulation of the mitochondrial pathway [60]. A consequence of this is that indirect cell killing through apoptotic mechanisms like Fas/FasL will destroy activated but uninfected cells while sparing the fraction of infected cells. Such a process will tend to increase the proportion of infected cells and diminish the proportion of uninfected cells in a tissue heavily burdened by SIV or HIV infection. Perhaps this mechanism contributes to the very high proportions of infected cells noted in macaque intestinal tissues after SIV infection [52] by removing uninfected cells and may help to reconcile apparent differences between direct and indirect cell depletion models.

Substantial data have been accumulated on HIV induction of Fas or FasL, the susceptibility of PBMC from HIV patients to apoptotic cell death and the reversal of these conditions by HAART. When viremia was suppressed by effective HAART, CD4 cells in PBMC had significantly reduced apoptosis that correlated with increasing CD4 counts in blood even though lymphoid tissue FasL levels were unchanged [61,62]. Similar findings have been reported for HIV and SIV infections. However, the problem remains that susceptibility to apoptosis that is measured *in vitro *rises and falls with changes in viremia, making it difficult to separate direct from indirect killing mechanisms in terms of their contribution to disease progression. In murine systems, these problems are addressed readily by the use of knock-out mutations eliminating Fas, FasL or both molecules. For AIDS-related questions, the initial studies are done most appropriately in nonhuman primates using SIV or SHIV to establish persistent infection and then applying interventions to modulate the Fas/FasL pathway.

Using a recombinant humanized anti-FasL monoclonal antibody [63] we developed a protocol for treating rhesus macaques to interrupt the Fas/FasL system. Animals received a total of 5 injections given once per week of anti-FasL beginning 2 weeks before SIV-inoculation and finishing 2 weeks after virus inoculation. The pilot study showed no effect of anti-FasL on plasma viremia, but found increased virus-specific immunity and delayed disease among treated animals [64]. A larger study using immunized macaques indicated that anti-FasL treatment preserved memory T cells and antigen responses after SIV infection, but was associated with decreased levels of Treg cells [65]. In the two interventional studies, anti-FasL delivered before and during the acute infection had a durable effect on immune status and disease many months after treatment stopped. These changes were not reflected in vRNA levels that were similar among treatment and control groups, but were detected in the composition and activity of other T cell populations. This means that uninfected effector cells must have been preserved by the treatment.

Another indication of the importance for indirect cell killing comes from studies of "naturally-infected" macaques i.e., sooty mangabeys infected with SIVsmm that maintain plasma viremia, do not show high susceptibility to *in vitro *apoptosis among PBMC and remain disease-free [66]. Here, apoptosis and immune activation were low and animals did not develop disease despite chronic viremia. The observation of preserved CD4 T lymphocytes with regenerative capacity in spite of high levels of viremia, suggests that virus alone cannot explain the massive loss of CD4 lymphocytes that occurs in pathogenic SIV and HIV infections [67] and indirect cell killing mechanisms may be important.

We reported [68] that half of the 56 macaques tested showed high levels of MHC-unrestricted cytolysis prior to SIV inoculation and that animals from this group were all rapid progressors. Subsequently, we found that MHC-unrestricted cytolysis involves the Fas/FasL pathway [64] and rapid progressors had higher baseline levels of cell killing through this pathway. In corroboration of these findings, high levels of lymph node cell apoptosis during acute SIV infection also predict that animals will become rapid progressors [69].

Reports showing lower levels of indirect cell killing/apoptosis among naturally-infected macaques despite similar viremia [64,67,70], the correlation between apoptosis and rapid progression [69] and intervention studies showing disease-sparing after brief treatment with monoclonal antibody against FasL [64,65]. These reports attest to strong relationships between apoptotic killing of uninfected cells and pathogenesis. The *in vitro *and tissue studies on HIV agree with these findings. Disagreements remain about the mechanisms for cell death, the roles for viral proteins and the relative importance of Fas versus non-Fas death pathways. In addition to direct infection and cell destruction by viral cytopathic effects or antigen-specific cytotoxicity, indirect cell killing mechanisms have broad impact on host immune capacity and are important in the pathogenesis of HIV/AIDS.

### Significance of indirect cell killing for HIV vaccine/therapeutics design

Knowing the role for uninfected bystander cell killing in disease, it remains a challenge to apply this information to treating HIV in man. An anti-Fas treatment during acute infection would be difficult to deliver. Since it may not modulate vRNA, the main marker for HIV therapy, lengthy studies would be needed to document treatment effects. The use of anti-retroviral therapy during this time would obscure the impact of blocking FasL. A combination of anti-FasL plus active immunization during interrupted HAART is conceivable, but unlikely to be pursued given the treatment choices and durable virus suppression achievable with available drugs. The most likely application of this knowledge may come in the evaluation of prophylactic or therapeutic vaccines. If we define viral proteins that are responsible for inducing bystander cell killing or identify particular motifs within these proteins that trigger killing mechanisms, vaccine-elicited antibodies may block these effects and preserve immunity even if vRNA levels appear unchanged. For example, gp41 peptide was shown to induce NKp44L on CD4+ T cells during HIV infection, making them highly susceptible to NK lysis. Immunizing against this peptide reduced ligand expression on CD4 lymphocytes and decreased apoptosis rates in SHIV-infected macaques [71] thereby indicating its role in promoting bystander cell killing. Careful evaluation of cellular apoptosis and tissue levels of Fas or FasL will be important to evaluate vaccine trials. FasL bearing CD4 lymphocytes were shown recently to kill antigen expressing cells following plasmid immunization, resulting in both lower antigen expression and subsequent decreases in antigen-specific cellular immunity [72]. Such mechanisms may impact other biological therapies in HIV/AIDS and appropriate inhibitors of Fas/FasL may be required to properly implement new interventions.

## Conclusion

HIV infection depletes a broad profile of leukocyte subsets including many that do not express CD4 and are not susceptible to direct virus infection. Models for the loss of non-CD4+ subsets including activation-induced cell death, explain the antigen-specific pattern of cell loss and frequently invoke Fas/FasL interactions as a principal mediator of cell death. The role for FasL was tested in the SIV/macaque model for AIDS using recombinant humanized monoclonal antibodies to neutralize this cell death ligand. Treated macaques modulated SIV disease and increased virus-specific immunity without consistent reduction in vRNA burden. The status of animals treated with anti-FasL was similar to natural SIVsmm infection of sooty mangabeys that have no overt disease despite high chronic viremia and show minimal apoptosis and immune hyperactivation.

The Fas/FasL death pathway is an important component of SIV or HIV disease. Whether this pathway for cell death is driven by viral proteins, virus particles or induced host factors remains unknown, although compelling examples of each exist in literature. The capacity to control activation of this cell death pathway may be important for the ultimate success of preventive vaccine strategies. The magnitude and duration of protective immunity, once viral exposure has occurred, are key to controlling infection and disease. Cell death pathways like Fas/FasL may be exploited by HIV to reduce the level of protective immunity and to establish a persistent infection with progressing disease. There is a continuing need to understand these mechanisms and develop effective interventions to improve the impact of antiretroviral therapy.

## Competing interests

The authors declare that they have no competing interests.

## Authors' contributions

PB wrote the manuscript and approved its content. CDP wrote the manuscript and approved its content. MSS wrote the manuscript and approved its content.
